# Effect of different sport environments on proactive and reactive motor inhibition: A study on open- and closed-skilled athletes *via* mouse-tracking procedure

**DOI:** 10.3389/fpsyg.2022.1042705

**Published:** 2022-12-12

**Authors:** Riccardo Bravi, Gioele Gavazzi, Viola Benedetti, Fabio Giovannelli, Stefano Grasso, Giulia Panconi, Maria Pia Viggiano, Diego Minciacchi

**Affiliations:** ^1^Department of Experimental and Clinical Medicine, University of Florence, Florence, Italy; ^2^Department of Neuroscience, Psychology, Drug Research and Child’s Health (NEUROFARBA), University of Florence, Florence, Italy; ^3^IRCCS SDN, Naples, Italy; ^4^Department of Physiology and Pharmacology “Vittorio Erspamer”, SAPIENZA University of Rome, Rome, Italy

**Keywords:** motor inhibition, proactive and reactive inhibitory control, sport training, open- and closed-skill sports, open- and closed-skilled athletes, mouse tracking, motor control, movement profiles

## Abstract

This study aimed to investigate the effect of different sport environments (open-and closed-skill sports) on proactive and reactive inhibitory processes as two distinct components of motor inhibition. A mouse-tracking procedure was employed to compare behavioral performance among three groups of participants (tennis players, swimmers and non-athletes) in non-sport-specific cued Go/No-Go (GNG) and Stop Signal Task (SST), which mainly engage proactive and reactive inhibitory control, respectively. Reaction times (RTs), inhibitory failures, and Stop Signal Reaction Times (SSRTs) were measured. To investigate dynamic aspects of inhibitory control, movement trajectories classified as one-shot (absence of trajectory alteration reflected in a steep slope) or non-one-shot (non-linear/multipeaked trajectory, with one or multiple corrections) were analyzed and compared among groups. Results showed no group differences in RTs in Go/No-Go and Stop conditions. SSRTs were significant shorter for the athletes than non-athletes in SST, but no differences emerged for inhibitory failures in cued GNG. During inhibitory failures athletes showed higher proportion of non-one-shot movements than non-athletes. Higher proportion of non-one-shot profiles was observed in cued GNG compared to SST. Finally, no differences between open-and closed-skilled athletes were found in both tasks. Our findings suggest that both proactive and reactive inhibitory controls do benefit from sport practice, but open-and closed-skill sports do not differ in influencing inhibitory processes. Movement profile analysis could be a promising, complementary behavioral analysis to integrate for more fine-grained evaluation and differentiation of inhibitory motor control in athletes, specifically when using GNG tasks.

## Introduction

In modern society, humans of all genders and ages engage deeply in sport activity, a practice grounded in the innate human capacity for play (Nesti, 2016). Sport environments provide a joyful source of entertainment, but also engender powerful perceptual-cognitive-motor experiences that promote the development of new skills and behavioral changes (Quarta et al., 2020).

The effects of sport training on cognitive motor control, which underlies the ability to coordinate thought and action to achieve adaptive goals (Banich et al., 2009), have received increased interest in the last years among neuroscientists (Yarrow et al., 2009; Walsh, 2014). A deep scientific knowledge of the effects of sports practice on cognitive functioning might have implications not only for talent identification and development in sport (Scharfen and Memmert, 2019) but also encourage specific disciplines as potential intervention for young and elderly populations with cognitive deficits (Tsai, 2009).

A key component of cognitive control functions associated with a flexible and efficient regulation of human behavior is the inhibitory motor control, defined as the ability to retain “prepotent” response tendency and suppress inappropriate ongoing actions (Duque et al., 2017). Inhibitory motor control is distinguishable in two distinct temporal dynamic components, termed as proactive and reactive motor inhibitory control (Aron, 2011; Meyer and Bucci, 2016). Proactive inhibition represents a top-down mechanism in which goal-relevant information is sustainably monitored over time to bias attention, perception, and action systems in order to optimize the inhibition of a planned action (Aron, 2011). Optimization of suppression of planned motor programs, before the occurrence of cognitively demanding external events, is strategic to avoid premature and inadequate movement tendencies and adapt behaviors to novel contextual information (Duque et al., 2017). Conversely, reactive inhibition is a “late corrective” process which is deployed when a planned or ongoing action needs to be canceled or interrupted outright in response to an external event (Aron, 2011). Proactive and reactive processes exert a synergic control on motor behavior and, as supported by previous functional neuroimaging literature, involve distinct but partially overlapped key brain regions (Meyer and Bucci, 2016; Gavazzi et al., 2020).

Inhibitory motor processes are essential for athlete’s performance and employed in a variety of sport context-related situations. For instance, for a successful shot in tennis, the athlete needs to proactively restrain the intended action until the “right time” by detecting relevant information coming from opponent’s stances. However, in case of an erroneous anticipation of the movement, it will be fundamental for the athlete to reactively stop the ongoing action and adapt to the updated circumstances.

Considerable evidence has shown the increased capacity of motor response inhibition in athletes, compared to non-athletes (i.e., Wang et al., 2013a; Bianco et al., 2017; Brevers et al., 2018; Heppe and Zentgraf, 2019), as well as athletes with higher-level expertise displaying a heightened inhibitory control than lower-level athletes (i.e., Huijgen et al., 2015; Verburgh et al., 2016). Noticeable are the results of Elferink-Gemser et al.’s study in which metacognition and executive functions (working memory, cognitive flexibility and inhibitory control) were assessed between competitive elite and sub-elite table tennis players. In this investigation table tennis players were found to score higher than the norm population on all tests, with elite players outperforming sub-elite table tennis players especially on the test for inhibitory control (Elferink-Gemser et al., 2018). In addition, a recent study revealed that high-intensity interval training generated temporary benefits on inhibitory control in experienced amateur boxers (Solianik et al., 2021). All together, these findings suggest that athletic performance is associated with inhibitory motor control efficiency and mechanisms underlying inhibition might be influenced and well benefit from exercise and sport training.

Recently, research efforts have been concentrated on evaluating the influences on cognitive functions exerted by different sport categories (i.e., Nakamoto and Mori, 2008; Di Russo et al., 2010; Wang et al., 2013a,b; Gu et al., 2019; Yu et al., 2019). The hypothesis that sport categories with different characteristics may differently influence cognitive functions of an individual is based on emergent concepts in neuroscience, including embodied cognition theories. According to these theories, cognition and perception features are forged by the kinds of experience that come from having a body interacting dynamically with the environment (Varela et al., 2016; Quarta et al., 2020).

To the extent of how bodily interaction with the surrounding environment can affect the performance of an athlete, sport disciplines have been classified into two macro-categories: open- and closed-skill sports (Singer, 2000). In open-skill sports, such as baseball, fencing or tennis, athletes are demanded to perform in a dynamically changeable, unpredictable, and externally-paced environment (Singer, 2000). At variance, closed-skill sports (e.g., track and field or swimming) are characterized by a relatively constant, predictable and self-paced environment (Singer, 2000). This broad classification, though is not completely exhaustive due to the nuances specific to a type of sport within each macro-category, allows to differentiate between sports, those in which goal-directed motor actions are continuously exposed randomly to accommodations to respond to the requirements of an environment characterized by a wide range of variations (i.e., open-skills), and others in which the form of learned movement sequencies is fairly fixed with a low level of dependency from the surrounding environment (i.e., closed-skills). As such, these requirements of movements within different sport contexts could impact differently on cognitive functions of practitioners. Hence, open-skill sport environments are expected to be more effective than closed-skill ones because of their inherently more demanding/stimulating nature. This assumption is suggested by evidence showing that exercise requiring complex, controlled, and adaptive movements may have an impact on cognitive control outcomes (Budde et al., 2008; Pesce et al., 2009; Best, 2010). Further support for open-skill contexts-induced superior cognitive benefits comes from recent physiological findings which showed in young adults a greater increase in serum BDNF after open-skill training compared with a closed-skill one (Hung et al., 2018). BDNF plays a critical role in neural plasticity and is considered as a biomarker of exercise-induced cognitive benefits (Poo, 2001; Huang et al., 2014). Accordingly, the greater BDNF changes resulted from open-skill exercise may also indicate its superior cognitive effect (Gu et al., 2019).

On the track of discovering the relation between open- and closed-skill sports and the development of cognitive functions, previous studies, which assessed the efficiency of inhibitory processes in athletes of these two categories, obtained inconsistent results. For instance, Nakamoto and Mori (2008) highlighted that baseball players outperformed gymnasts and track and field athletes only during the execution of a baseball-specific experimental task and not in a non-sport-specific one. Conversely, Wang et al. (2013a) reported a higher level of inhibitory motor control in open-skilled tennis players than closed-skilled swimmers during a non-sport-specific inhibitory task. Given a relatively few number of studies and results obtained so far, there is the need for further research investigating the role of open- and closed-skill sports backgrounds in affecting inhibitory control and the potential effect of extensive specific sport practice in developing cognitive adaptations outside the specific sport context (Furley and Memmert, 2011). In this sense, the present study investigated the differences in proactive and reactive inhibitory control between athletes and non-athletes as well as between athletes from two different sport categories (i.e., tennis and swimming). To serve this goal, cued Go/No-Go (GNG) task and Stop Signal Task (SST) with non-sport-specific design were used to assess the capacity to inhibit an action.

It is noteworthy that the GNG task and the SST are typical experimental paradigms which, though often used interchangeably (Meyer and Bucci, 2016), might investigate different components of the motor inhibitory functioning (Raud et al., 2020). In fact, the GNG mainly elicits preparatory processes for the inhibition of motor behavior, namely before the appearance of an imperative stimulus, reflecting the active maintenance of task goals (Verbruggen and Logan, 2008). In contrast, the SST mainly involves the reactive processes as an already initiated motor response is required to be canceled after an imperative signal occurrence (i.e., stop signal; Ray Li et al., 2008).

Also, in the GNG and the SST, the behavioral indices commonly used to assess inhibitory performance are the commission errors rate in inhibiting action and the Stop Signal Reaction Time (SSRT), respectively. In the GNG, commission errors rate can be estimated by the number of failed actions implemented during No-Go trials, in which inhibition is required. Instead, the SSRT is a measure representing the time required to withhold the prepotent, ongoing action in response to the “stop” signal and it is calculated based upon the independent race model (Logan and Cowan, 1984). This model formalizes inhibition as a “race” between a process of response, triggered by the presentation of a Go stimulus, and a stopping process, activated by the presentation of a stop signal. Short SSRT reflects an efficient inhibitory control.

The main modality used in several inhibitory studies to compute behavioral performance in GNG and SST involves the press of a key (Wilkowski and Robinson, 2016). However, given its dichotomic nature, this approach is prone to shortcoming because it can allow to record only the “success” or “failure” responses, omitting any interactions that occur during the decision-making process (Leontyev et al., 2018). Indeed, recent evidences suggested that motor inhibition should be studied as disruptive processes rather than in an all-or-none fashion (Leontyev et al., 2018; Leontyev and Yamauchi, 2019; Nguyen et al., 2021). The exploration on how the nervous system exerts flexibility to inhibit a prepotent response or suppress inappropriate ongoing actions could give an insight into the dynamic nature of motor inhibition processing.

In this perspective, the characterization of hand movement trajectories by mouse-tracking procedures appears as a potentially valuable methodology to analyze deeply the dynamic aspects of inhibitory control (Freeman and Ambady, 2010). Movement trajectories are classified as one-shot or non-one-shot based on their profiles. One-shot movements are characterized by the absence of trajectory alteration reflected in a steep slope, while non-one-shot movements are considered when there is the presence of one or multiple corrections (Morasso, 1981; Flash and Hogan, 1985; Fishbach et al., 2005, 2007). Motor control theories suggest that a point-to-point planned movement has no trajectory corrections unless there is an occurrence of specific control processes during movement preparation (Soechting and Lacquaniti, 1981; Atkeson and Hollerbach, 1985). As such, a non-one-shot movement profile could reflect the interference of proactive and reactive mechanisms during a planned motor response.

This methodology has been used in a recent study by Benedetti et al. (2020) to characterize movement profiles related to proactive and reactive inhibition during cued GNG and SST in a population of healthy subjects. Authors assessed either when subjects correctly responded in the Go conditions or when they failed to inhibit responses in No-Go/Stop conditions of both tasks. They found a higher proportion of non-one-shot movements in the cued GNG compared to the SST when individuals failed to inhibit responses and suggested that proactive component may be responsible for unsmooth profiles in inhibition failures (Benedetti et al., 2020).

In the current study, we employed for the first time a behavioral method based on a mouse response-registration system to investigate the effect of open- and closed-skill sport practice on proactive and reactive inhibition during non-sport-specific cued GNG and SST. Considering previous research studying this issue (Nakamoto and Mori, 2008; Wang et al., 2013a), a population of male athletes was enrolled for this study along with a control group of male non-athletes.

Tennis players and swimmers were selected as representatives for open- and closed-skill categories. In general, we predicted a superiority of athletes’ groups than non-athletes in performing both cued GNG (lower commission errors rate in inhibiting action) and SST (shorter SSRTs). Moreover, as practice of open-skill activity requires continuous complex cognitive operations to coordinate and constantly adapt nonautomated gross motor actions to ever-changing demands of the unpredictable environment (Best, 2010), we hypothesized that tennis players would perform better than swimmers and non-athlete controls. Finally, by introducing a one-shot and non-one-shot movement profiles analysis, the methodology proposed in this study gave the opportunity to explore whether and how the practice of sport and different sport categories would influence dynamic aspects of inhibitory control.

## Materials and methods

### Participants

A total of 47 male healthy young adults participated in the study. We chose to recruit all male participants to make a consistent link with the previous literature in which male populations were enrolled to investigate how different open- and closed-skill sports practice affect motor inhibition in young adult athletes (Nakamoto and Mori, 2008; Wang et al., 2013a). The final sample size was based on Wang et al.’s study (*η*^2^*_p_* = 0.29). To achieve a Power (1 - beta) higher than 0.90 with effect size *f* = 0.63 (*η*^2^*_p_* = 0.29) and alpha = 0.05, we computed (G*Power 3.1.9.7; Faul et al., 2007) a sample size of at least 14 participants per group.

So, our sample included 15 tennis players (open-skilled group, aged 20.80 ± 3.05 years; with experience of 11 to 22 years) and 16 swimmers (closed-skilled group, aged 21.13 ± 3.16 years; with experience of 12 to 24 years) affiliated to the Italian Tennis Federation (FIT) and the Italian Swimming Federation (FIN), and 16 non-athletes (control group). Tennis group consisted of singles players with all of them self-reporting to play commonly a limited number of doubles matches during each prior and current seasons. Swimmers group consisted of 9 freestyle, 2 butterfly, 2 breaststroke, 1 backstroke and 2 individual medley swimmers (Gerard et al., 1986). In this study tennis players and swimmers players who competed at national or regional level were included. Top-level athletes who were internationally competitive, who had trained to compete at the internationally levels but never qualified to do so, or belong to national teams, were excluded (Huang et al., 2017). All athletes were recruited from different tennis and swimming sport clubs of central Italy. The remaining 16 healthy subjects belonging to control group were a subsample selected from a larger cohort as those examined in a previous study (Benedetti et al., 2020), mainly recruited from the students’ community of the University of Florence. The selected participants were males and they reported no currently or previously engagement in sport activity at a competitive level, no historical specialty in any sport/exercise, and were inactive at the time of the study (aged 24.38 ± 3.40 years). Participants were predominantly right-handed except six left-handed (2 tennis players, 2 swimmers, and 2 non-athletes) according to the laterality score from the Edinburgh Handedness Inventory (Oldfield, 1971). The level of physical activity (i.e., kilocalorie expenditure) (Sallis et al., 1985; Wang et al., 2013a) and the aerobic fitness (VO_2_max index; mL*kg^−1^*min^−1^) were also assessed for each participant (Bradshaw et al., 2005; Wang et al., 2013a). The level of physical activity was assessed by employing the 7-day physical activity recall questionnaire (Sallis et al., 1985), a survey which was shown to objectively quantify the total energy (kcal) expenditure over a seven-day period (Maximova et al., 2009). The aerobic fitness was assessed by adopting a non-exercise formula using descriptive subjects’ characteristics (age, sex and BMI), as well as the Physical Activity Rating (PA-R) questionnaire (George et al., 1997; Wang et al., 2013a), and the Perceived Functional Ability (P-FA) questionnaire (George et al., 1997; Wang et al., 2013a). This formula was shown to provide a good prediction of the actual VO_2_max (*R* = 0.93) in adults aged from 18–65 years (Bradshaw et al., 2005), and can be used to estimate the state of athletes’ fitness (Chan et al., 2011). The formula is described as follows: VO_2_max (mL *kg^−1^ *min^−1^) = 48.073 + (6.1786 × Sex; female = 0, male = 1) – (0.2466 × Age) – (0.6196 × BMI) + (0.7126 × P-FA) + (0.6716 × PA-R). Table 1 summarizes the subjects’ characteristics.

**Table 1:** Characteristics of tennis players (open-skilled group), swimmers (closed-skilled group) and non-athletes (control group).

	Tennis players (*n* = 15)	Swimmers (*n* = 16)	Non-athletes (*n* = 16)
Age (years)	20.80 ± 3.10^#^ (range 18–29)	21.13 ± 3.16^#^ (range 18–28)	24.38 ± 3.40 (range 18–30)
Height (*m*)	1.81 ± 0.10	1.78 ± 0.07	1.81 ± 0.07
Weight (Kg)	72.10 ± 9.91	73.50 ± 7.97	82.56 ± 11.78
BMI (Kg/m^^2^)	21.90 ± 2.20^#^ (range 18–24)	23.13 ± 1.51^#^ (range 22–24)	25.10 ± 3.08 (range 18–29)
Years of experience	15.00 ± 3.02 (range 11–22)	15.69 ± 3.36 (range 12–24)	/
Age started playing sport	5.80 ± 1.26 (range 4–7)	5.44 ± 1.59 (range 4–8)	/
hours a day of practice	3.23 ± 1.27 (range 2–6)	3.09 ± 0.66 (range 2–4)	/
days a week of practice^Δ^	4.87 ± 0.92 (range 3–6)	5.93 ± 0.26 (range 5–6)	/
Kilocalorie expenditure (Kcal/day)	3967.73 ± 862.02^#^	3845.93 ± 923.01^#^	2848.79 ± 311.47
Kilocalorie expenditure (Kcal/week)	27773.99 ± 6034.14^#^	26921.50 ± 6461.21^#^	19941.53 ± 2180.31
VO_2_max (mL*kg^−1^*min^−1^)	58.42 ± 2.32^#^	57.68 ± 2.08^#^	41.16 ± 3.65

Values are shown as mean ± SD; # denotes significant differences from non-athletes (*p* < 0.05); Δ denotes significant differences between tennis players and swimmers (*p* < 0.05).

Participants were naive to the task and blinded to the purpose of the study. They did not receive a compensatory fee for participation. In addition, all tested individuals reported having normal or corrected-to-normal visual acuity, and no history of motor or neurological impairments or cardiovascular diseases. Also, they were not taking any medications that could affect cognitive functions at the time of the experiment. The study protocol was performed according to the Declaration of Helsinki requirements and approved by the institutional ethics committee (Prot. N. 0027365). All participants gave written informed consent.

### Experimental procedure

The experimental session took place in a quiet room partially devoid of natural light and with no artificial light to obtain a semi-darkness environment. For the athletes, it was made certain that the testing session was not performed during or after one of their daily training sessions (Brevers et al., 2018).

All participants underwent an interview and completed the 7-day physical activity recall questionnaire together with aerobic fitness questionnaires.

**Figure 1:**
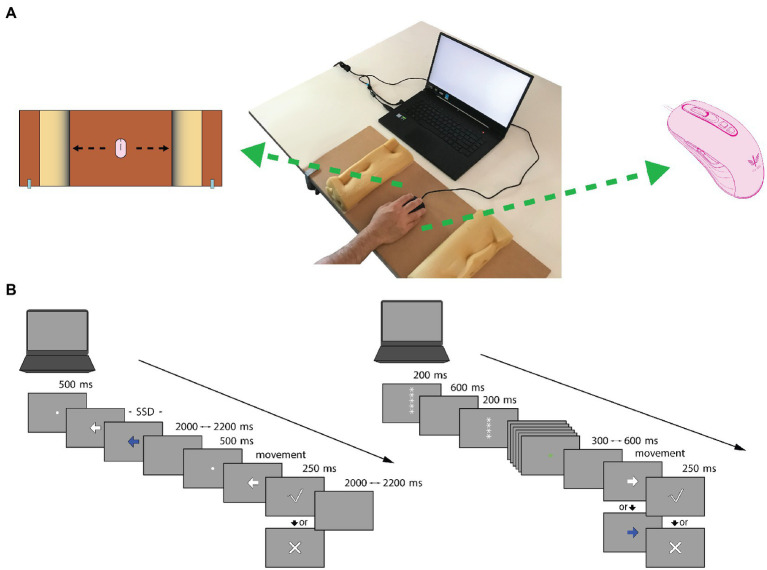
Mouse tracking system Setup and Experimental paradigms. **(A)** Mouse tracking system Setup. On the center: the system consists of a 15-inch laptop placed on a table with a mouse device positioned in the center of a wooden board. On the left: to emit a response, participants were instructed to move the mouse, from the center, in parallel to the x-axes of the board as quickly and accurately as possible in the direction indicated from the Go-stimulus (i.e., left or right), until they reached the barrier, bumping against it. After the response, the mouse had to be put back in the center. To inhibit a response, participants were instructed to not move the mouse from the center of the board. On the right: optical gaming mouse-peripheral (KEY IDEA, model G10S). **(B)** Experimental paradigms. Trial structure of the cued Go/No-Go (GNG) is presented on the right. Each trial started with a descending series of white five asterisks, with the latter three asterisks changing color to provide information about the probability that a “Go-stimulus” might occur (green asterisks = high ‘Go-stimulus’ probability, red asterisks = low ‘Go-stimulus’ probability). Subsequently, the target stimulus appeared, and participants had to emit or inhibit (white arrow = emit, blue arrow = inhibit) the motor response. After correct responses in Go-stimulus trials, feedback on response speed was provided (check mark = sufficient speed, X mark = too slow). Trial structure of the Stop Signal Task (SST) is presented on the left. A white arrow was presented at the beginning of each trial, and participants were instructed to emit a response. Only on a minority of trials, after a variable amount of time, named stop-signal delay (SSD), a blue arrow appeared and participants were instructed to stop the motor action, overriding the previous instruction. After correct responses in Go-trials, feedback about response speed was given, as for the cued GNG.

Once this phase was completed, participants performed the cued GNG (Gavazzi et al., 2019; Benedetti et al., 2020) and the SST (Logan and Cowan, 1984; Ray Li et al., 2008). Each participant was tested individually, sitting on a comfortable chair at a distance of 57 cm from a 15-inch laptop placed on a table (Figure 1A). The order of two behavioral tasks was randomized across participants to obtain a balanced number of subjects who performed first the cued GNG or the SST. Each task was immediately preceded by a training session to familiarize participants with experimental procedures. It consisted of five sample trials for each task.

For both tasks, motor responses were acquired using an optical gaming mouse peripheral (KEY IDEA, model G10S, dimensions 4.84 × 2.64 × 1.53; weight 136 g; Figure 1A) placed on the center of a wooden board (a cross mark was used as reference point) delimited by two 28 × 10 cm sponges. Each sponge was positioned at 12.5 cm from the cross mark of the wooden board. The wooden surface measured 30 × 70 cm and was anchored to the table by metal clamps to ensure stability (Figure 1A). Visual stimuli production and response recording were obtained using OpenSesame 3.2.6 Kafkaesque Koffka (Mathôt et al., 2012).

In both tasks all participants performed movements with their right hand, regardless of their handedness, as the six left-handed subjects enrolled for the study reported the preferential daily use of the right hand for a mouse device.

### Cued Go/No-Go

Target visual stimuli consisted of left- or rightward pointing arrows (4 cm × 4 cm, ~4° of visual angle) displayed in the center of the computer screen (Figure 1B). Starting from the middle of the wooden board (cross mark), participants were instructed to emit a motor response by moving the mouse as quickly and accurately as possible in the direction indicated by a “Go” visual target stimulus (50% of trials) until they impacted the sponge barrier (Figure 1A); conversely, they were required to withhold the response when a visual “No-Go” target stimulus was presented. The “Go-stimulus” and “No-Go-stimulus” were colored white and blue, respectively (Figure 1B). Both of them disappeared when the sponge barrier was impacted or once 1,000 ms was passed.

Target stimuli were always preceded by a descending series of asterisks which was presented as a countdown at the beginning of each trial. This procedure of visual cueing was employed to prepare the participant for the proper target stimulus and to enhance the proactive preparatory phase (Giovannelli et al., 2016; Gavazzi et al., 2019)_._ Each trial started with five white asterisks and the countdown continued until only one asterisk appeared on the screen. On every appearance, asterisks remained on screen for 200 ms and a 600 ms blank interval followed in between. The color of the asterisks changed from the third appearance during the countdown and provided information on the probability that a “Go-stimulus” might be presented. Namely, green asterisks indicated a “high probability” in which Go-stimulus was likely to occur (70%; 56 trials), whereas red asterisks indicated a “low probability” in which Go-stimulus was likely to occur (30%; 24 trials). Participants were informed about the association between asterisk colors and the “high” or “low” Go stimulus probabilities. The total time of the countdown was 3,400 ms. The time between the end of the countdown and the appearance of the target stimulus varied randomly between 300 and 600 ms.

For “Go-stimulus” trials the first asterisk of the following trial was presented immediately after the feedback elapsed (see below in *Feedback on Response Speed* paragraph); for “No-Go-stimulus” trials it was presented immediately after the maximum response time elapsed for correctly inhibited responses and after the threshold was reached for erroneous responses (Gavazzi et al., 2019).

The task consisted of 160 trials that presented the four possible cue-target combinations: green asterisks and Go-stimulus; green asterisks and No-Go-stimulus; red asterisks and Go-stimulus; red asterisks and No-Go-stimulus. The order of “Go-stimulus,” “No-Go-stimulus” and relative asterisks countdown trials was randomized for each participant.

### Stop signal task

SST included two types of trials, “Go-trials” and “Stop-trials” (Figure 1B). Each of them began with a 500-ms white fixation point placed at the center of the computer screen to engage participants’ attention. Following the offset of the fixation point, target visual stimuli consisting of arrows (4 cm × 4 cm, ~4° of visual angle) appeared centrally to the screen.

In Go-trials (70% of trials), a white arrow (go-stimulus) pointed randomly toward left or right (56 left-arrow and 56 right-arrow trials). Participants had to move as quickly and accurately as possible the mouse in the direction indicated by the arrow until they impacted the sponge barrier (Figure 1A). In Stop-trials (30% of trials), the white arrow was replaced, after a variable delay, by a blue arrow (stop-signal) pointing in the same direction, to which participants were instructed to withhold or to suppress the on-going motor response. The blue arrow disappeared after 1,000 ms or as soon as the participant failed to inhibit a motor response (i.e., responses in which the mouse reached the sponge barrier).

The stop-signal delay (SSD) is the temporal interval between the occurrence of the go-stimulus and the stop-signal. The ability to stop a motor response is a function of the length of the SSD: the longer the SSD is, the more difficult it is to stop a motor response (Sharp et al., 2010). SSD was adjusted in steps of 50 ms across the course of the SST based on the participant’s performance by using a 1-up/1-down standard adaptive tracking procedure (Brevers et al., 2018; Verbruggen et al., 2019; Benedetti et al., 2020): if participant inhibited successfully in a stop-trial, the following SSD got 50 ms longer and the task got harder; conversely, the following SSD was shortened by 50 ms when the participant failed to inhibit the motor response, making the next trial easier (Verbruggen et al., 2019). This procedure guaranteed that participants have an inhibition success rate on approximately 50% of stop-signal trials and that each of them was tested around the individual threshold (Verbruggen et al., 2019). The starting SSD value was individually set based on a 20-trial simple choice reaction time (CRT) test performed by each participant at the beginning of the experimental phase. Therefore, in the first trial of the SST, the SSD was the mean response time obtained at the CRT test minus 200 ms (Sharp et al., 2010). The order of the Go-trials and Stop-trials was randomized for each participant. The task consisted of a total of 160 trials. The inter-trial interval was varied randomly between 2,000 and 2,200 ms.

### Feedback on response speed

In both tasks, feedback on the response speed was given to limit the slowing tendency which can be adopted by the participant as a strategy to improve accuracy (Sharp et al., 2010). Namely, negative feedback (white X mark) was displayed for response trials with slow reaction times and participant was instructed to speed up the response in the subsequent trial. Conversely, when participant reached the sponge barrier within a pre-set time, a check mark (positive feedback) appeared on the screen. The feedback remained on the screen for 250 ms. The maximum response time after which the negative feedback was provided was the mean response time obtained at a simple CRT test minus one standard deviation. This procedure allowed the use of a stringent but realistic time response threshold reflecting individual differences in the speed of processing and response (Sharp et al., 2010). The feedback was provided only on response speed for “Go-conditions” (i.e., Go-stimulus trials of the cued GNG and Go-trials of the SST). Participants did not receive feedback on their performance accuracy in terms of correct or erroneous responses.

### Data analysis

All subjects’ characteristics were described using means and standard deviations (SDs), and group differences were evaluated for age, BMI, VO_2_max and kilocalorie expenditure (per day and per week) using an one-way ANOVA with SPORT CATEGORY as between-subject factor (three levels: tennis players, swimmers, and non-athletes). Also, years of experience, age started playing sport, hours a day and days a week of practice were compared between tennis players and swimmers by means of independent samples t-tests (two tailed).

### Response execution and RT

A response was considered as implemented once the individual exceeded a pre-determined threshold value (i.e., 30 pixels from the starting position) and a mouse shift within 30 pixels was considered a device error. This value was chosen on the basis of preliminary recordings performed to calibrate the experimental apparatus (Benedetti et al., 2020). More precisely, one experimenter (V.B.) had to hold the mouse (230 Dots Per Inch —sampling rate 500 Hz) trying to stay as still as possible for 5 min. The farthest value obtained on the x-axis by the mouse was registered. After this, in another session lasting 5 min, the same experimenter had to keep the mouse still while alternating this condition with some random mouse movements. The farthest x values reached during stillness interval were recorded. The maximum value measured during the two sessions was 30 pixels (3 mm) and it was chosen as threshold to prevent “false positive” movements. For both tasks, all mouse movements exceeding this threshold value were considered either as correctly implemented responses or as inhibitory failures.

Once response execution was established, RT was measured as the time between the target stimulus appearance and the mouse movement onset. Particularly, mouse movement onset was considered as the moment when mouse device passed 4.5 pixels on the x-axis in order to achieve higher timing accuracy.

### Behavioral performance

Behavioral performance in cued GNG and SST was firstly quantified by the following measures: number of correct responses and reaction times (RTs) in the Go-conditions (Go-stimulus trials and Go-trials for GNG and SST, respectively), number of inhibitory failures (commission errors rate) and RTs in the No-Go/Stop conditions (i.e., No-Go trials and Stop-trials, respectively).

For the cued GNG, correct responses and inhibitory failures were calculated as total and as a function of the Go-stimulus probability (low and high probability). In this task, where the proactive control is mainly engaged, the proportion of inhibitory failures (i.e., the proportion of No-Go trials in which subjects failed to stop) was considered a measure of inhibition efficiency.

For the SST, where the reactive component is enhanced, inhibition efficiency was assessed by the inhibition latency which was estimated by computing Stop Signal Reaction Time (SSRT) index through the mean method (see Verbruggen et al., 2019 for details).

To evaluate the overall behavioral performance, the proportion of correct responses as well as RTs in the Go-conditions were entered in a mixed analysis of variance (ANOVA) with SPORT CATEGORY as between-subject factor (three levels: tennis players, swimmers, and non-athletes) and TASK as a within-subject factor (three levels: cued GNG “high Go-stimulus probability,” cued GNG “low Go-stimulus probability,” and SST).

Additionally, commission errors rates during high and low Go-stimulus probability conditions of the cued GNG were entered in a mixed ANOVA with SPORT CATEGORY as between-subject factor (three levels: tennis players, swimmers, and non-athletes) and TASK as within-subject factor (two levels: “high Go-stimulus probability”, cued GNG “high Go-stimulus probability”).

Moreover, for the SST specifically, RTs were entered in a mixed ANOVA with SPORT CATEGORY as between-subject factor (3 levels: tennis players, swimmers, and non-athletes) and CONDITION as within-subject factor (two levels: Stop-trials and Go-trials) to ensure that SST performance met the assumptions of the independent race model (Logan and Cowan, 1984) which predicts that failed Stop-trials RTs will be faster than Go-trials RTs. Also, SSRTs were calculated for each subject and entered in a one-way ANCOVA with SPORT CATEGORY as between-subject factor (three levels: tennis players, swimmers, and non-athletes) while controlling for age and BMI as covariates. Finally, the overall performance in the SST was further described through the inhibition function (which plots the probability of inhibition failures as a function of the SSD) (Logan and Cowan, 1984). The correlation between the proportion of inhibition failures and SSD values was tested as well.

Effect sizes were calculated as either partial eta squared (*η*^2^*_p_*) or Cohen’s *d* based on the analysis. Post-hoc pairwise comparisons were conducted by Fisher’s least significant difference (LSD) tests. A *p* value <0.05 was considered significant.

### Movement profiles

**Figure 2:**
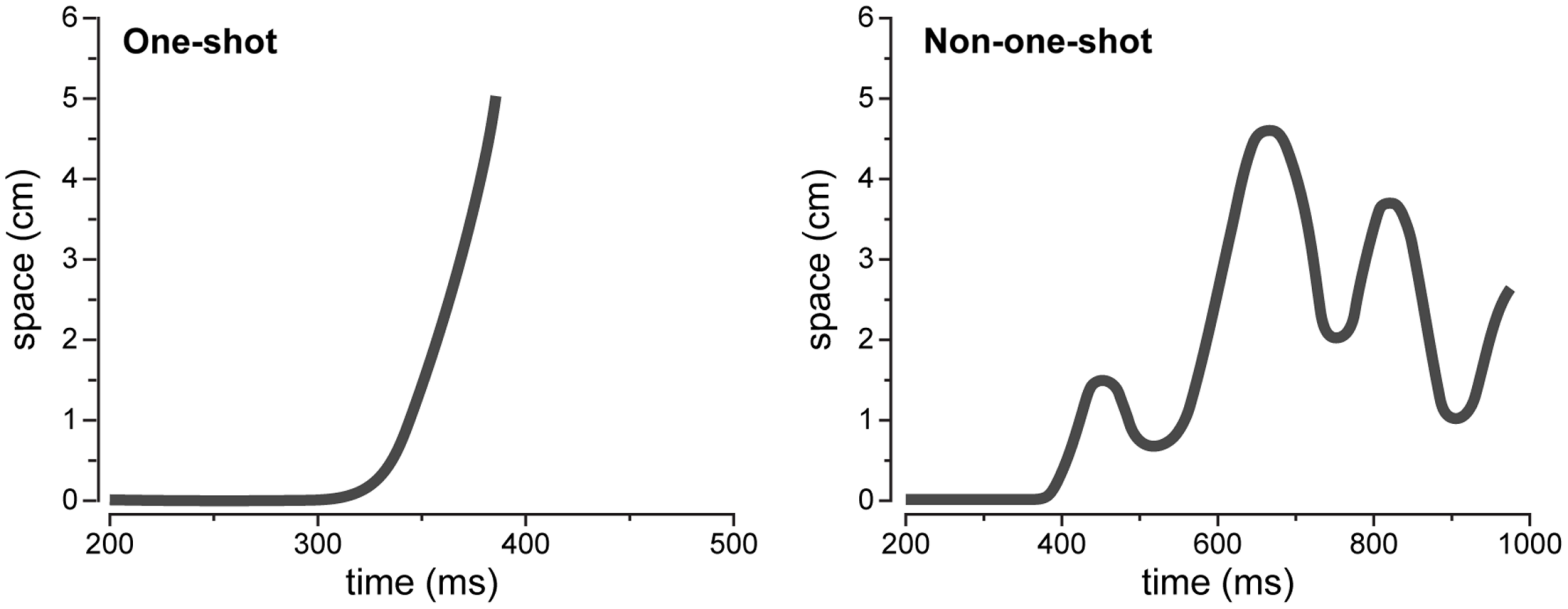
Movements profiles from one representative subject. A sample of one-shot movement profile is presented on the displacement-time graph on the left. A sample of non-one-shot movement profile is presented on the displacement-time graph on the right.

In both tasks we evaluated movement profiles of subject responses extrapolated from mouse trajectories both for correct responses obtained in the Go-conditions and inhibitory failures of the No-Go/Stop conditions. We classified movements as one-shot or non-one-shot based on their profiles. Particularly, one-shot movement was characterized by the absence of trajectory alteration reflected in a steep slope, without any peak, as depicted in the Time-Displacement graph (see Figure 2, on the left). This profile reflects a smooth movement without motor command alteration (Morasso, 1981; Flash and Hogan, 1985). In contrast, non-one-shot movement was characterized by non-linear/multipeaked trajectory, with one or multiple corrections (see Figure 2, on the right). A non-one-shot profile reflects alteration over the initial motor plan (Fishbach et al., 2005, 2007). A MATLAB custom script was used to automate the movement profiles classification; the code was firstly validated by matching automated results with visually inspected and manually categorized profiles.

Given the dichotomic nature of the dependent variable such as one-shot vs. non-one-shot profiles, differences between independent variables (SPORT CATEGORY: 3 levels, tennis players, swimmers and non-athletes; TASK: 3 levels, cued GNG “high Go-stimulus probability,” cued GNG “low Go-stimulus probability” and SST) in relation to movement profiles were detected using a generalized linear model with binomial distribution and logit link (Factorial ANOVA - GLZ; Statsoft 14). Age and BMI were included in the model as covariates. A Chi-square post-hoc analysis was then applied with Holm-Bonferroni correction to interpret significant interactions.

## Results

### Groups characteristics

Table 1 shows the characteristics of the three groups.

Significant groups differences for age were found [*F*_(2,44)_ = 5.978, *p* = 0.005, *η*^2^*_p_* = 0.270]. Post-hoc analysis showed a significantly lower mean age in tennis players and swimmers compared to non-athletes (*p* = 0.002, *d* = 1.252 and *p* = 0.011, *d* = 1.146, respectively) but no significant differences between the two groups of athletes (*p* = 0.541, *d* = 0.105).

There were significant groups differences for BMI [*F*_(2,44)_ = 6.080, *p* = 0.005, *η*^2^*_p_* = 0.247]. Both tennis players and swimmers showed a significantly lower BMI values as compared to non-athletes (*p* = 0.001, *d* = 1.264 and *p* = 0.036, *d* = 0.900, respectively), whereas no significant differences were observed between the athletes of different sport categories (*p* = 0.125, *d* = 0.665).

Also, significant differences among groups were shown for VO_2max_ [*F*_(2,44)_ = 149.317, *p* < 0.001, *η*^2^*_p_* = 0.890]. VO_2max_ values in tennis players and swimmers were found to be significantly higher when compared to non-athletes (*p* < 0.001, *d* = 6.000 and *p* < 0.001, *d* = 6.056, respectively), while no differences were shown between tennis players and swimmers (*p* = 0.433, *d* = 0.335).

Moreover, for the estimated levels of physical activity per day and per week, significant groups differences emerged [per day: *F*_(2,44)_ = 6.094, *p* = 0.005, *η*^2^*_p_* = 0.248; per week: *F*_(2,44)_ = 6.095, *p* = 0.005, *η*^2^*_p_* = 0.248]. Post-hoc analysis showed significantly higher kilocalorie expenditure in tennis players (per day: *p* = 0.002, *d* = 1.570; per week: *p* = *0*.002, *d* = 1.570) and swimmers (per day: *p* = 0.005, *d* = 1.299; per week: *p* = 0.005, *d* = 1.299) compared to non-athletes. No differences between swimmers and tennis players were displayed (per day: *p* = 0.676, *d* = 0.136; per week: *p* = 0.676, *d* = 0.136).

Finally, no significant differences between tennis players and swimmers were found for years of experience [*t*_(29)_ = 0.445, *p* = 0.660, *d* = 0.160], age started playing sport [*t*_(29)_ = −0.291, *p* = 0.773, *d* = 0.105], and hours a day of practice [*t*_(29)_ = 2.007, *p* = 0.054, *d* = 0.721], whereas there was a significant difference for days a week of practice [*t*_(29)_ = 4.508, *p* < 0.001, *d* = 1.620], with swimmers having a higher number of days of practice per week.

### Behavioral performance

Details on performance in cued GNG and SST are given in Table 2.

**Table 2:** Behavioral performance for both cued GNG and SST.

		Cued GNG			SST
		Total	High-Go	Low-Go	
Go-conditions					
Tennis players	Correct responses	0.95 ± 0.06	0.94 ± 0.07	0.96 ± 0.06	0.94 ± 0.07
	RT	382 ± 28	377 ± 28	392 ± 32	393 ± 54
Swimmers	Correct responses	0.94 ± 0.07	0.94 ± 0.07	0.95 ± 0.07	0.96 ± 0.04
	RT	367 ± 31	361 ± 33	379 ± 33	367 ± 54
Non-athletes	Correct responses	0.97 ± 0.04	0.97 ± 0.04	0.96 ± 0.06	0.97 ± 0.03
	RT	383 ± 33	378 ± 31	394 ± 39	392 ± 49
No-Go/Stop conditions					
Tennis players	Inhibitory failures	0.02 ± 0.02	0.03 ± 0.03	0.02 ± 0.02	0.69 ± 0.13
	RT	369 ± 54	365 ± 47	377 ± 53	371 ± 34
Swimmers	Inhibitory failures	0.04 ± 0.04	0.06 ± 0.07	0.04 ± 0.04	0.71 ± 0.11
	RT	370 ± 53	356 ± 55	365 ± 59	356 ± 49
Non-athletes	Inhibitory failures	0.03 ± 0.04	0.05 ± 0.06	0.03 ± 0.04	0.66 ± 0.11
	RT	390 ± 39	366 ± 41	397 ± 35	365 ± 45

Correct responses values are reported in proportion and mean reaction times (RTs) are reported in ms. All values are shown as mean ± SD. GNG, Go/No-Go; High-Go, High Go-stimulus Probability; Low-Go, Low Go-stimulus Probability; SST, Stop Signal Task.

Task performance in the Go-conditions of cued GNG and SST was highly accurate, as revealed by the proportion of correct responses, with no significant main effect for TASK [*F*_(2,88)_ = 0.595, *p* = 0.554, *η*^2^*_p_* = 0.013] or SPORT CATEGORY [*F*_(2,44)_ = 0.609, *p* = 0.548, *η*^2^*_p_* = 0.027] or interaction [*F*_(4,88)_ = 0.602, *p* = 0.662, *η*^2^*_p_* = 0.027].

From RTs analysis during Go-conditions of cued GNG and SST, a significant main effect for TASK [*F*_(2,88)_ = 5.275, *p* = 0.007, *η*^2^*_p_* = 0.107] emerged. Namely, RTs were significantly faster for the “high Go-stimulus probability” condition (372 ms ± 31 ms) compared to the “low Go-stimulus probability” condition (388 ms ± 34 ms) of the cued GNG (*p* < 0.001, *d* = 0.866), whereas no significant differences were found between “high Go-stimulus probability” condition and SST (383 ± 52 ms) and between “low Go-stimulus probability” condition and SST (*p* = 0.053, *d* = 0.293 and *p* = 0.488, *d* = 0.106, respectively). No significant main effect was found for SPORT CATEGORY [*F*_(2,44)_ = 1.516, *p* = 0.231, *η*^2^*_p_* = 0.064] with no significant interaction [*F*_(4,88)_ = 0.307, *p* = 0.873, *η*^2^*_p_* = 0.014].

In addition, for the cued GNG, no significant main effect of TASK [*F*_(1,44)_ = 3.834, *p* = 0.057, *η*^2^*_p_* = 0.080] or SPORT CATEGORY [*F*_(2,44)_ = 1.189, *p* = 0.314, *η*^2^*_p_* = 0.051] or interaction [*F*_(2,44)_ = 0.994, *p* = 0.378, *η*^2^*_p_* = 0.043] on commission errors rate was found, with athletes of different sport categories and non-athletes showing comparable low numbers of inhibitory failures in both “high Go-stimulus probability” and “low Go-stimulus probability” conditions of No-Go trials.

For the SST, a significant main effect of CONDITION on RTs [*F*_(2,44)_ = 26.235, *p* < 0.001, *η*^2^*_p_* = 0.374] was found, with failed Stop-trials RTs being faster than Go-trials RTs (*p* < 0.001, *d* = 0.741). By contrast, no significant main effect of SPORT CATEGORY on RTs [*F*_(2,44)_ = 0.884, *p* < 0.420, *η*^2^*_p_* = 0.039] and no significant interaction [*F*_(2,44)_ = 1.330, *p* < 0.275, *η*^2^*_p_* = 0.057] were revealed. These results indicated that for all three groups the performance met the assumptions of the independent race model. Accordingly, SSRTs were calculated for all groups.

**Figure 3:**
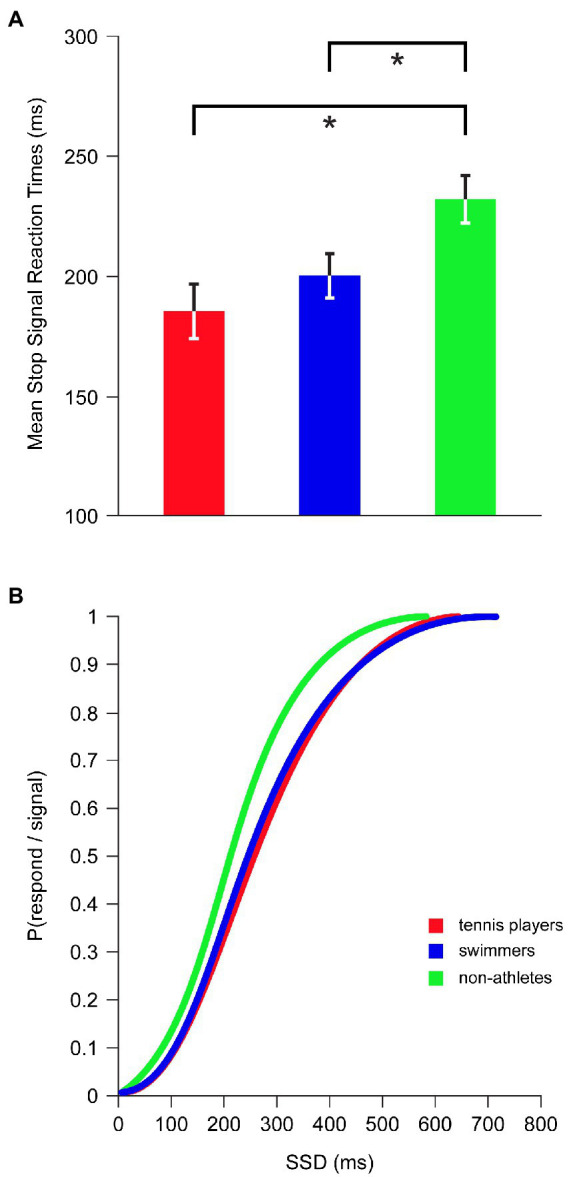
Inhibitory performance in SST across tennis players, swimmers and non-athletes. **(A)** Mean Stop Signal Reaction Times. Error bars represent ±1 standard error of the mean. Asterisk marks a significant difference, *p* < 0.05. **(B)** Inhibition function: P(respond/signal) at a given SSD were collapsed for each group and fitted through a Weibull curve using maximum likelihood methods.

Inhibition latency, indexed by the SSRT, showed a significant main effect for SPORT CATEGORY [*F*_(2,42)_ = 4.981, *p* = 0.011, *η*^2^*_p_* = 0.192] while controlling for age and BMI (Figure 3A): tennis players (185 ± 45 ms; *p* = 0.003; *d* = 1.106) and swimmers (201 ± 35 ms; *p* = 0.021; *d* = 0.825) had significantly shorter SSRTs compared to non-athletes (232 ± 40 ms), indicating a superior inhibitory control in athletes who were faster at inhibiting a prepotent ongoing motor response than non-athletes. No significant differences were found between tennis players and swimmers (*p* = 0.258; *d* = 0.400).

As expected by the inhibition function, the proportion of inhibition failures was significantly increased with the increment of SSD (*r* = 0.356, *p* < 0.001). The inhibition functions of tennis players and swimmers were shifted to the right with respect to non-athletes’ inhibition function (Figure 3B), reflecting an overall superiority in inhibition efficiency of both categories of athletes, relative to non-athletes, as the task difficulty increased.

### Movement profiles

The proportion, mean and standard deviation of the binomial distribution of one-shot movements, calculated for either Go or No-Go/Stop conditions for both tasks, are given in Table 3.

**Table 3:** One-shot profiles for both the cued GNG and SST.

	Cued GNG			SST
	Total	High-Go	Low-Go	
Go-conditions				
Tennis players	0.99; 1,106 ± 3	0.99; 770 ± 3	0.99; 335 ± 2	0.99; 1,488 ± 4
Swimmers	0.99; 1,170 ± 3	0.98; 805 ± 4	0.99; 356 ± 2	0.99; 1,633 ± 4
Non-athletes	0.98; 1,177 ± 5	0.98; 826 ± 4	0.99; 354 ± 2	0.99; 1,647 ± 4
No-Go/Stop conditions				
Tennis players	0.56; 15 ± 3	0.37; 3 ± 1	0.63; 12 ± 2	0.76; 363 ± 9
Swimmers	0.41; 22 ± 4	0.48; 10 ± 2	0.37; 12 ± 3	0.81; 436 ± 9
Non-athletes	0.65; 28 ± 3	0.56; 10 ± 2	0.72; 18 ± 2	0.87; 428 ± 7

Proportion, mean and standard deviation of the binomial distribution of one-shot movements, calculated for either Go or No-Go/Stop conditions. GNG, Go/No-Go; High-Go, High Go-stimulus Probability; Low-Go, Low Go-stimulus Probability; SST, Stop Signal Task.

For Go-conditions, the analysis on movement profiles did not show any main effect of SPORT CATEGORY [Wald test *χ*^2^ (2) = 0.072, *p* = 0.964) or TASK (Wald test *χ*^2^ (2) = 2.220, *p* = 0.329] or interaction [Wald test *χ*^2^ (4) = 3.276, *p* = 0.513].

On the contrary, for No-Go/Stop conditions, the analysis on movement profiles, while controlling for age and BMI, revealed significant main effects of both SPORT CATEGORY [Wald test *χ*^2^ (2) = 6.285, *p* = 0.043] and TASK [Wald test *χ*^2^ (2) = 42.13, *p* < 0.001] whereas the interaction between these two factors was not significant [Wald test *χ*^2^ (4) = 5.386, *p* = 0.250].

**Figure 4:**
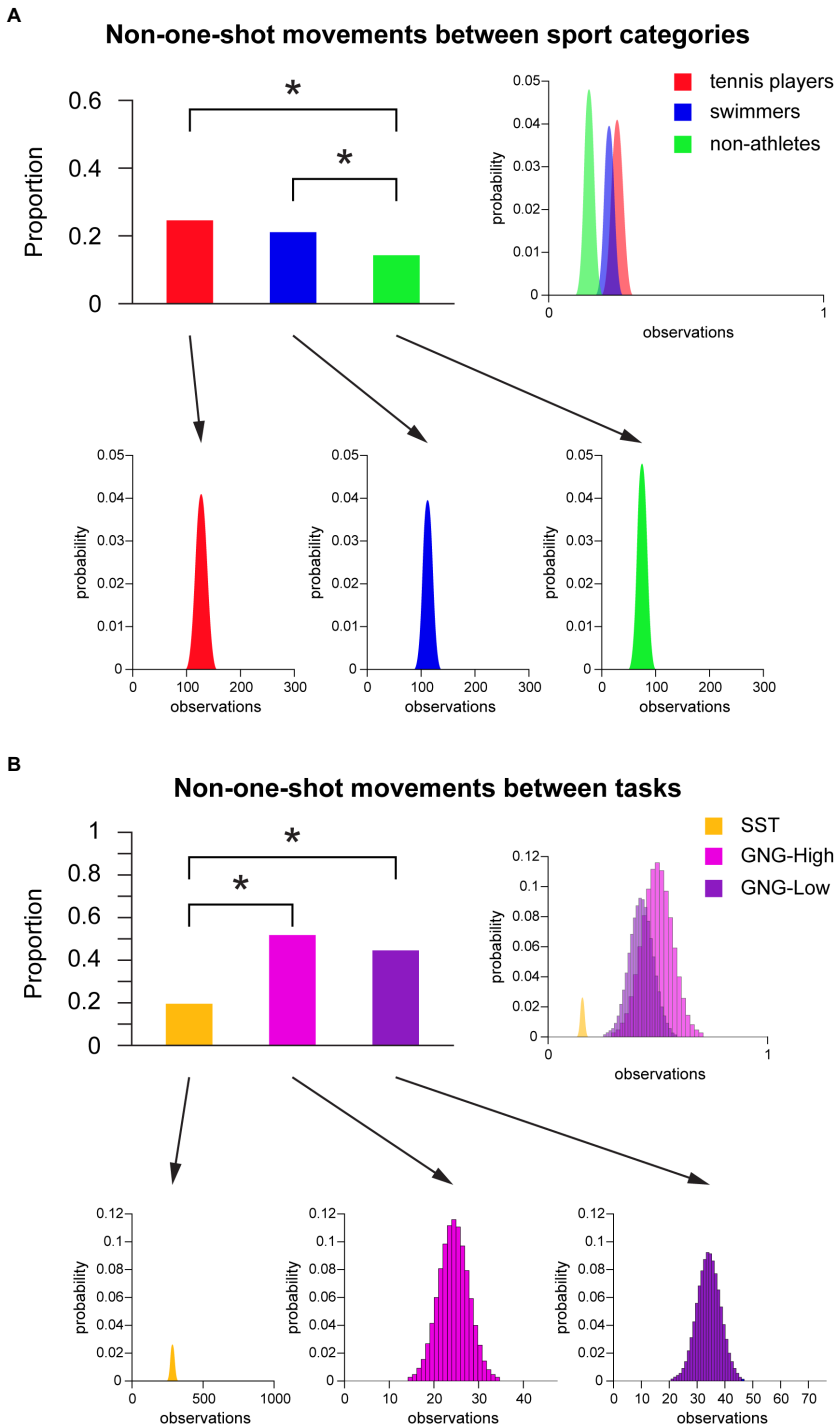
Non-one-shot movements. **(A)** Non-one-shot movements between sport categories regardless the task. Upper (on the left): Bar graph of non-one-shot movement proportion of tennis players, swimmers and non-athletes. Asterisk marks a significant difference, *p* < 0.05. Upper (on the right): Superimposed binomial distribution for each group, normalized for number of observations. Lower: Binomial distribution for each group. **(B)** Non-one-shot movements between tasks regardless the sport category. Upper (on the left): Bar graph of non-one-shot movement proportion of SST, cued GNG-High condition, and cued GNG-Low condition. Asterisk marks a significant difference, *p* < 0.05. Upper (on the right): Superimposed binomial distribution for each task, normalized for number of observations. Lower: Binomial distribution for each task. Abbreviations: GNG = Go/No-Go; High-Go = High GO-stimulus Probability; Low-Go = Low GO-stimulus Probability; SST = Stop Signal Task.

Post-hoc Chi square analysis revealed that, during inhibitory failures, tennis players and swimmers showed a significantly lower proportion of one-shot profiles compared to non-athletes regardless the task. Complementarily, a higher proportion of non-one-shot movements was found for both categories of athletes compared to non-athletes (Figure 4A). Specifically, tennis players showed a significantly higher proportion of non-one-shot movements compared to non-athletes [Wald test *χ*^2^ (1) = 16.43, *p* < 0.001, Holm-Bonferroni adjusted]. Swimmers showed a significantly higher proportion of non-one-shot movements compared to non-athletes as well [Wald test *χ*^2^ (1) = 11.89, *p* = 0.002, Holm-Bonferroni adjusted], whereas no significant differences emerged between tennis players and swimmers [Wald test *χ*^2^ (1) = 0.549, *p* = 0.459, Holm-Bonferroni adjusted] (Figure 4A).

Moreover, post-hoc analysis revealed that the proportion of one-shot movements was higher in the SST compared to the cued GNG “high Go-stimulus probability” [Wald test *χ*^2^ (1) = 30.403, *p* < 0.001, Holm-Bonferroni adjusted] and “low Go-stimulus probability” [Wald test *χ*^2^ (1) = 30.944, *p* < 0.001, Holm-Bonferroni adjusted] conditions, with consequently a significantly higher proportion of non-one-shot movements in these latter two conditions (Figure 4B). However, there were no significant differences between “high” and “low” Go-stimulus probability conditions in the cued GNG [Wald test *χ*^2^ (1) = 0.467, *p* = 0.495 Holm-Bonferroni adjusted] (Figure 4B).

## Discussion

In the current study, we investigated how different open- and closed-skill sports practice affect proactive and reactive motor inhibition evaluated by non-sport-specific cued GNG and SST paradigms. A mouse-tracking procedure was employed for the first time in athletes in order to explore the dynamic aspects of inhibitory processes.

Our results indicated that athletes exhibit better reactive inhibitory control compared to non-athletes, as revealed by the performance in the SST. In contrast, no differences between athletes and non-athletes emerged in the cued GNG task. The analyses of the movement profiles suggested that, although the accuracy was similar, the performance of athletes could rely on proactive inhibitory control to a greater extent compared to non-athletes. Taking into account that complex cognitive operations are required for nonautomated motor actions to adapt to unpredictably changing environments, we expected that open-skilled athletes would outperform closed-skilled athletes. Our findings did not confirm this hypothesis. Indeed, behavioral performance and movement profiles did not differ between tennis players and swimmers, as representatives for open- and closed-skill sports respectively, in both cued GNG and SST.

To the best of our knowledge, very few studies have explored both proactive and reactive inhibition in athletes (Bianco et al., 2017; Brevers et al., 2018) and in no case have movement profiles been characterized. For instance, in the study by Brevers et al. (2018), a modified version of the SST was used: cues were inserted to inform subjects about the probability of an upcoming stop-signal to evaluate proactive and reactive components. However, it is difficult to clearly separate at behavioral level these two complementary processes within the same task (Benedetti et al., 2020). So, keeping in mind the aim to better evaluate each process and to maximize the link between movement profile analysis and the respective inhibitory control component mainly involved in each task, two separate paradigms were used.

Using SST to focus on the reactive component of inhibitory processes, we found that both tennis players and swimmers exhibited shorter SSRTs than non-athletes. The SSRT estimates the covert latency of the stop process (Logan and Cowan, 1984). Based on the independent race model, response inhibition depends on competition between two processes: the Go- and the Stop-response processes (Logan and Cowan, 1984). A successful inhibition necessitates the latter process to “win the race.” Accordingly, a shorter SSRT denoted a superiority in reactive inhibition efficiency in athletes who were faster at inhibiting a prepotent motor response than non-athletes.

Non-converging results emerged from studies employing the SST as paradigm to evaluate differences in inhibitory efficiency between athletes and non-athletes and between players of different expertise (Wang et al., 2013a; Chen et al., 2019; Heppe and Zentgraf, 2019; Meng et al., 2019). For instance, Meng et al. (2019) found no differences of SSRT values between open-skilled athletes (i.e., volleyball and badminton players) and sedentary controls. Also, Chen et al. (2019) tested the effects of long-term badminton training in response inhibition and re-engagement using SST and change-signal task, respectively. Authors showed that no differences between badminton players and sedentary controls were reported during SST and argued that SST did not fit properly for examining inhibitory control between open-skilled athletes and non-athletes as the other task (Chen et al., 2019). Our findings contrast with the abovementioned studies.

In the SST, the successful stop can be influenced by response slowing strategies (Verbruggen et al., 2019). For this reason, we considered the feedback as an important factor which could influence the final result. Therefore, with application of feedback, we built a stringent and time constraint design minimizing the use of response slowing strategies. According to this, RTs in go trials obtained by our participants were considerably faster than those reported in most of other studies investigating the role of sport in influencing inhibitory processes and using a SST without feedback (Chen et al., 2019; Meng et al., 2019). In fact, in a study by Wang et al. (2013a) in which there was the employment of an auditory feedback on speed response, RTs were quite similar to those of our groups, and differences in SSRTs between open-skilled athletes (i.e., tennis players) and non-athletes were also observed. However, unlike Wang’s work (2013a), we did not observe SSRT differences between open-skilled and closed-skilled athletes. Differences in results appear to be related to the equally higher performance of tennis players and swimmers tested in our study. Indeed, when comparing the SSRTs between the two studies, it was observable that similar values were obtained by non-athletes, whereas the values for both two groups of athletes, especially for swimmers, were lower in our investigation than Wang’s. This equally higher efficiency in performing SST in our open- and closed-skilled athletes compared to non-athletes was also confirmed by the inhibition function (see Figure 3B). According to this, the inhibition functions of tennis players and swimmers were shifted to the right with respect to non-athletes’, indicating an overall superiority in inhibition efficiency of both category of athletes as the task difficulty increased.

A close observation in the characteristics of our participants may contribute to explain the lack of differences between tennis players and swimmers, a result in contrast with Wang’s study in which the superiority in inhibitory motor control of open-skilled athletes was shown (Wang et al., 2013a). While in Wang’s study a sport practice experience ranging from 5 to 6 years was reported, athletes enrolled for the present study had an average experience of 15 years (range 11–24 years). Given that there are evidences that physical and sport activities are a strong gene modulator which determine structural and functional changes in the brain and induce benefit on cognitive functioning (Mandolesi et al., 2018), it is plausible that the longer physical training is, the stronger efficiency in cognitive function could develop. To this regard, a study evaluating the influence of training experience and skill level on judo athletes revealed that the senior group with longer training experience had higher cognitive performance than the junior group, suggesting that the length of sport training could influence cognitive functions capacity, though the results might also be dependent on the types of sport (Supinski et al., 2014). Moreover, a recent study on the longitudinal physical activity-cognition relationship captured long-term changes and associations between physical activity and cognitive function which may support the hypothesis that inhibitory motor control benefits more from longer experience in sport training (Stenling et al., 2021). Future longitudinal and cross-sectional studies are strongly required to evaluate the effect of long-term sports practice relevant to the types of sport on modulating inhibitory function development.

An alternative explanation for the similar results obtained by two sport categories could be related to the sport starting age of the athletes (with an average age of: 5–6 years old). It is well known that the most intensive development of all components of executive functions, including inhibitory control, takes place during early and mid-childhood, usually between 6 and 12 years of age (Best, 2010; Bidzan-Bluma and Lipowska, 2018). In a meta-analysis of studies on executive functions from age 5 to adulthood, Romine and Reynolds (2005) reported the greatest growth in inhibition of prepotent responses from the age of 5 to 11 years old. Following this stage, inhibitory mechanisms continue to develop, although at a decreased rate, until adolescence period from which no age-related increase is displayed (Romine and Reynolds, 2005). This might be the result of differences in the age-related maturation of prefrontal cortex. During early and mid-childhood, the prefrontal cortex grows and develops very rapidly, making it particularly prone to take influences from exercise interventions (Anderson, 2002; Bidzan-Bluma and Lipowska, 2018). Sport experience during such maturation stages, when in prefrontal cortex neural substrate corresponding to inhibitory control is developing, might have prompt the well exploitation and growth of this cognitive capacity and the relative neural circuits (Mandolesi et al., 2017). Therefore, with both swimmers and tennis players starting specific sport activities from the age of 5 to 6 years old, it is conceivable that they might benefit more from sport practice and have a better efficiency in inhibitory motor control compared to athletes with older sport starting age (see Wang’s study; Wang et al., 2013a). Based on the above considerations, we suggest that inhibitory processes development should be considered correlated with not only the sport category but also the years of experience and starting age, especially when the sport interventions start at critical stage of development, such as in our study.

Regarding the cued GNG task, no differences in the behavioral performance were observed between athletes and non-athletes, and between tennis players and swimmers, as revealed by comparable no-go commission errors rates. The GNG is one of the most commonly used paradigms to evaluate inhibitory processes in clinical populations, as well as sport experts. Commission errors rate was reported as a critical behavioral parameter to effectively differentiate populations with a variety of neuropsychiatric disorders from healthy subjects (e.g., Woltering et al., 2013). In contrast, when employed in individuals who have specific talents or skills, such as sport experts, the failures rate tended to show remarkably small values (Chiu et al., 2020; (Nakamoto and Mori, 2008; Wang et al., 2013b; Yamashiro et al., 2015, 2021). For instance, an EEG study by Nakamoto and Mori (2008) reported an augmented P300 amplitude in baseball players (open-skills) in comparison with gymnastics and track and field athletes (closed-skills) during No-Go trials of a GNG. The authors interpreted these results as a proof of the superior inhibitory efficiency in baseball players. However, the errors rate parameter showed very low values (around 1%) and did not evidence differences in behavioral performance between the two groups of athletes (Nakamoto and Mori, 2008). Similar results were obtained by Wang’s study (2013b) which used the GNG variable foreperiod paradigm characterized by several conditions adopted by Nakamoto and Mori (2008) to investigate the effects of different sporting practice on nonspecific temporal preparation. In the same line, Yamashiro et al. (2015), using a somatosensory GNG to investigate the effects of specific athletic training regimens on sensorimotor inhibitory processes, did not show significant differences between athletes of different sport categories in errors rate, though they found significant differences in neural correlates being related to inhibitory mechanisms.

In the attempt to prevent this near floor effect in the commission errors, in the present study, feedback was introduced on the response speed at the end of each Go-stimulus trial. This is in connection with the notion that quick decisions are more error prone while accurate ones take longer time (speed-accuracy trade-off) (Bogacz et al., 2006). Nevertheless, despite the implementation of this strategy, commission errors rates in both athletes and non-athletes were relatively low. Therefore, putting together our results and those of abovementioned studies, it could be inferred that commission error rate of the GNG paradigm is not sensitive enough to unveil behavioral performance differences related to proactive inhibitory efficiency when tested healthy individuals or sport experts.

Interestingly, while errors rate index failed to differentiate the groups, movement profiles analysis revealed differences between athletes and non-athletes during inhibitory failures. Tennis players and swimmers showed a lower proportion of one-shot movements (which complementarily resulted in a higher proportion of non-one-shots) compared to non-athletes. Again, no differences were observed between two sport categories.

The distinctive smooth and straight trajectory of one-shot profile is typical of rapid point-to-point hand movements, regardless of the direction or amplitude, indicating that no factors influence preparation and execution phases of the original motor plan (Morasso, 1981; Flash and Hogan, 1985; Bullock and Grossberg, 1988). Therefore, one-shot profile during failures to withhold responses in No-Go/Stop conditions might suggest that action monitoring and inhibitory processes are not successfully intervening on movement planning and implementation.

Conversely, the unsmooth and multipeaked trajectory of non-one-shot profile likely reflects adjustments in the implementation of the initial motor plan. Non-one-shot movement profile often characterizes hand movements under stringent constraints of time and spatial accuracy (Milner and Ijaz, 1990; Novak et al., 2002). Trajectory modifications were interpreted as corrections of motor commands (Fishbach et al., 2005, 2007). These irregularities were considered either as a result of a continuous controller interacting with the environment (Shadmehr and Mussa-Ivaldi, 1994; Bhushan and Shadmehr, 1999), or as evidence for a controller that iteratively generates discrete, corrective submovements in response to a mismatch between the state of limb monitoring and the desired endpoint (Meyer et al., 1988; Fishbach et al., 2005). On these bases, we hypothesize that non-one-shot profile during inhibitory failures might reflect an attempt of action control mechanisms to correct the erroneous movement which does not correspond to the task goal. Alternatively, it might indicate the competition between conflicting motor tendencies of going and stopping in condition of uncertainty about the type of response. The higher proportion of non-one-shot movements for athletes, therefore, appears to unveil the connection between extensive sport practice and a task goal-related performance during inhibitory protocols. Sport training might foster mechanisms of action control over inhibitory failures as well as render the subject more prone to adhere to cognitive demands of the current environment.

It should be noted that movement profiles also differed between tasks during No-Go and Stop conditions. The proportion of non-one-shot profiles was found significantly higher in the cued GNG (hence in the mainly proactive task) compared to the SST (mainly reactive task). This is in line with findings obtained in a previous study by Benedetti et al. (2020) on a sample of healthy subjects with no experience in competitive sport activity. In this study, by using the same experimental procedure, movement profile analysis showed that inhibitory failures in the cued GNG conditions were more frequently associated with non-linear/multipeaked movement trajectories with respect to those in the SST. According to the prevalent inhibitory component engaged by the cued GNG task employed, the authors proposed that non-one-shot profiles might be associated with proactive inhibition. Considering that proactive control promotes the maintenance of task goals to optimally bias action systems in a goal-driven manner (Aron, 2011), it was hypothesized that inhibitory failures characterized by non-one-shot profiles, which were predominant under proactive circumstances, could reflect that proactive control might prompt mechanisms dedicated to movement monitoring over erroneous movement. An alternative hypothesis was that non-one-shot profiles reflect conflicting motor tendencies due to interactions between proactive inhibition and action readiness. Accordingly, given the fact that there was an equally higher proportion of non-one-shot movements in open- and closed-skilled sport experts compared to non-athletes in our results, it could be interpreted that athletes seem to rely more on proactive strategy than non-athletes to withhold the action. Proactive mechanisms are strongly resource consuming, and athletes might be able to more easily sustain proactive control during action inhibition than non-athletes, which might explain the higher proportion of non-one-shot movements compared to non-athletes. However, keeping in mind that present data did not show differences in the behavioral errors rate index between athletes and non-athletes when performing the cued GNG, our interpretation should be considered speculative and further investigated in future studies.

Altogether, whereas sport practice appeared to be beneficial for enhancing inhibitory motor functioning, our findings cannot support the hypothesis according to which cognitively demanding and nonautomated environments - typical of open-skill disciplines - can be superior in influencing the development in response inhibition. Even though results obtained from non-one-shot movements analysis should still be taken with caution, it is plausible to suggest that both open-and closed-skilled athletes showed no significant differences in proactive and reactive inhibitory control. Our results might challenge previous studies where open-skill sport practice had been documented to be superior in modulating motor inhibition (Wang et al., 2013a; Formenti et al., 2021). However, we are indeed in line with a very recent study which detected no differences between elite open-skilled and closed-skilled sport experts in inhibition using a modified Eriksen flanker task (Koch and Krenn, 2021).

Some limitations concerning the current investigation should be acknowledged. The sample size of the three groups remains relatively small. We only tested young males and further studies are needed in order to verify whether these results are generalizable to young females (Bianco et al., 2020). Moreover, in our study we employed a cross-sectional design, which allowed to only investigate the correlation whether the practice of a type of sport influences motor inhibition. Longitudinal studies should be of much interest to better analyze the influence of sport practice over a long period, also considering other factors (i.e., innate predispositions) that have been shown to correlate with cognitive performance (Wei et al., 2019). Finally, we studied proactive and reactive inhibitory processes as two components of motor inhibition using two dedicated tasks. However, since these components are distinct but not completely separated from each other, the association between non-one-shot movement analysis and the respective inhibitory control component mainly involved in each task remains uncertain and further studies are required to better investigate this aspect. With these considerations, we suggest that additional electrophysiological and brain imaging methods could be adopted to accompany with movement profiles analysis for the better understanding of the dynamic nature of inhibitory functioning.

## Conclusion

The current study provided an insight into how involvement in open- and closed-skill sports modulates proactive and reactive inhibitory processes as two distinct components of motor inhibition using non-sport-specific cued GNG and SST. A mouse-tracking procedure was introduced to conduct for the first time a one-shot and non-one-shot movement profiles analysis in order to investigate the dynamic aspects of inhibitory control on athletes. According to our findings, though inhibitory control does benefit from sport practice, the superiority of open-skill settings in modulating inhibitory control is not obvious and the question whether or not and to what extent different sport environments would influence motor inhibition seems to be under debate. To better explore this issue, future studies should consider the interaction between other factors (i.e., years of sport experience and sport starting age) and different sport environments to influence the development of inhibitory functioning. This could take a step forward in understanding how sports with different modalities can be used as potential strategic intervention to evolve inhibition.

## Data availability statement

The raw data supporting the conclusions of this article will be made available by the authors, without undue reservation.

## Ethics statement

The studies involving human participants were reviewed and approved by Ethical Committee of the University of Florence. The patients/participants provided their written informed consent to participate in this study.

## Author contributions

RB: study conceptualization, methodology, investigation, data curation, data interpretation, conception for figures design, writing original draft, writing – review and editing, and funding acquisition. GG: methodology, data curation, formal analysis, data interpretation, and writing – review and editing. VB: investigation, data curation, formal analysis, data interpretation, and writing – review and editing. FG: study conceptualization, methodology, data interpretation, writing – review and editing, and funding acquisition. SG: data curation, data interpretation, and writing – review and editing. GP: data curation, data interpretation, and writing – review and editing. MPV: study conceptualization, methodology, and writing – review and editing. DM: study conceptualization, methodology, conception and art work for figures of manuscript, and writing – review and editing. All authors contributed to the article and approved the submitted version.

## Funding

This work was supported by the grant for “Bando di ateneo per il finanziamento di progetti competitivi per ricercatori a tempo determinato (RTD) dell’Università di Firenze – Anno 2021–2022,” University of Florence.

## Conflict of interest

The authors declare that the research was conducted in the absence of any commercial or financial relationships that could be construed as a potential conflict of interest.

## Publisher’s note

All claims expressed in this article are solely those of the authors and do not necessarily represent those of their affiliated organizations, or those of the publisher, the editors and the reviewers. Any product that may be evaluated in this article, or claim that may be made by its manufacturer, is not guaranteed or endorsed by the publisher.
